# A comparison of methods to measure central and peripheral oxytocin concentrations in human and non-human primates

**DOI:** 10.1038/s41598-017-17674-7

**Published:** 2017-12-08

**Authors:** Arthur Lefevre, Raphaëlle Mottolese, Manon Dirheimer, Carmine Mottolese, Jean-René Duhamel, Angela Sirigu

**Affiliations:** 1 Institut des Sciences Cognitives Marc Jeannerod, Centre National de la Recherche Scientifique, UMR 5229 Bron, France; 2 Université Claude Bernard Lyon 1, Lyon, France; 3 Neurosurgery Unit 500, Hôpital Pierre Wertheimer, Hospices Civils de Lyon, Lyon, France

## Abstract

Oxytocin (OT) concentration in the blood is considered to be a marker of its action in the brain. However, two problems have emerged when measuring OT level in the blood. First, it is unclear whether different methods of assessment lead to similar OT values. Second, it is unclear if plasma OT concentrations is informative on what OT does in the brain. To clarify these issues, we collected cerebrospinal fluid (CSF) from the brain ventricle of 25 patients during surgery to compare with plasma OT after simultaneous blood withdrawal. Additionally, we collected 12 CSF and blood samples from non-human primates while awake or under anaesthesia. We used four methods to assay OT concentrations: Commercial EIA with/without extraction, laboratory developed EIA with filtration and RIA with extraction. Three of these methods showed a positive correlation between plasma and CSF OT, suggesting a link between plasma and central OT, at least under specific testing conditions. However, none of the methods correlated to each other. Our results show major disagreements among methods used here to measure peripheral and brain OT and therefore they call for more caution when plasma OT is taken as a marker of central OT.

## Introduction

Oxytocin (OT) is a neuropeptide synthesized in the hypothalamus which has a wide range of actions both in the brain and in the body^1^. Notably, OT is implicated in various aspects of social behaviour and can positively or negatively modulate how an individual interacts with conspecifics^2^. This OT functional property has attracted a lot of attention within the scientific community, hoping to develop new pharmaceutical approaches to treat psychiatric disorders such as autism or schizophrenia^3^, which are characterized by social dysfunction. In this context, one way to study OT in humans is to measure its concentration in the plasma and to correlate it with a behavioural outcome^4^. Although widely used this approach suffers of two major flaws. Firstly, it is unclear whether peripheral oxytocin (OTp) is linked to central oxytocin (OTc). To demonstrate such a link, researchers have tried to compare plasma OT concentrations to OT levels in the cerebrospinal fluid (CSF), the latter thought to reflect the global activity of the oxytocinergic system in the brain^5^. Secondly, there is an important debate regarding how to measure OT levels, since it is unclear whether different assessing methods can generate converging results.

Anatomical evidence shows that OTp and OTc are released through independent pathways. The paraventricular (PVN) and the supraoptic (SON) nuclei of the hypothalamus, the centres of OT synthesis, contain magnocellular neurons with axons projecting to the posterior pituitary, where OT is released into the bloodstream^5^. OT is distributed in extracellular space and CSF mainly through dendritic release^6,7^. Axonal and dendritic release of OT seem to be independently regulated^8^ and do not act on the same time scale: axonal release is instantaneous while dendritic release is slower (OT peaks in CSF dozens of minutes after stimulation)^9^. Despite evidence against a coordinated action between brain and body OT, yet some animal studies suggest the existence of a link. Wotjak and colleagues found that in chronically stressed rats plasma OT concentrations reflected OT levels in the SON^10^, thus suggesting that although independent, OTc and OTp can be released simultaneously. Further, Knobloch *et al*.^11^ discovered a group of PVN magnocellular neurons projecting both to the amygdala and to the posterior pituitary, releasing OT in these two regions. These results were confirmed recently by studies showing central projections of OT neurons, notably an axonal magnocellular pathway in the forebrain^12–14^. Following these results, it is reasonable to conclude that, under certain conditions, there is a coordinated axonal release of OT in specific brain areas and in the blood.

Whether similar mechanisms regulate OT release in humans is unknown. Previous attempts to link OTp and OTc have led to divergent results. Although two studies performed in headache patients and in children have found a correlation between OTp and OTc^15,16^ four others failed to find a significant link^17–20^. In all of these experiments, CSF OT was collected by doing a lumbar puncture, a method that extracts CSF in a region far from OT synthesis site. OT collected at this remote place can be degraded compared to brain OT because CSF flow is slower in the spinal cord^7^. Moreover, lumbar puncture procedure interfere with neurotransmitters levels including OT^21,22^, by generating acute stress, a condition that further modulates OT concentrations^10,23^. This view finds support in a recent meta-analysis which shows that the relationship between central and peripheral levels of OT is dependent on sampling condition^24^.

In addition to the lack of knowledge regarding the biological significance of OTp, serious methodological criticisms have recently emerged concerning its measurements^25,26^. Before the advent of commercial Enzyme Linked-Immunosorbent Assay (EIA), the most common approach was the Radioimmunoassay (RIA) technique associated with solid phase extraction. Several laboratories have developed this technique in the eighties and many studies have obtained physiologically plausible results. Thus, RIA has been since considered as the gold standard for assessing peripheral OT. More recently, plasma concentrations of OT have been measured with the immunoassay method combined with an extraction process to remove interfering proteins. With this procedure, baseline plasma OT levels are typically comprised between 1–10 pg/mL^25^. When several teams started to assay non-extracted samples, their results conducted to extremely high values. For instance OT levels can go from 1.8 pg/mL after extraction up to 358 pg/mL without extraction^27^. High values obtained without extraction are problematic because they do not match the theoretical concentration when considering OT production rate, release and clearance rate^26^. Hence, OT pre-processing whether performed with solid phase extraction or filtration is strongly recommended in order to obtain robust results on plasma OT real concentrations. Thus, we believe it is urgent to conduct a comparison between the methods employed to investigate plasma OT.

The aim of this work was to study the correlation between OTc and OTp and to compare different sample pre-processing techniques (extraction, filtration, none) and type of assay (commercial EIA, laboratory EIA^28^ and RIA^18^).

We investigated central and peripheral oxytocin by collecting brain CSF and blood in patients undergoing brain surgery. This method allows direct access to central CSF within the space of the third ventricle, the subarachnoid cavity or from a ventriculoperitoneal shunt tap. Samples were tested using three methods (commercial EIA, laboratory EIA and RIA).

In addition, to look at the effects of anaesthesia and to test a fourth method, the most used one (commercial EIA with solid phase extraction), we collected simultaneous CSF and blood samples from awake or anaesthetized macaques.

## Material and Methods

### Human experiment

#### Participants

Twenty-five adult patients (12 men and 13 women, mean age 40.6, ±14.5, range 20–81) requiring brain surgery (Neurological and Neurosurgical Hospital in Lyon) participated in this study. Patients with history of severe depression or other major psychiatric disorders, those abusing of drug, alcohol and/or tobacco, and pregnant women were excluded from the study.

All patients or their legal guardian gave a written informed consent. The study was approved by the Ethical Committee Lyon Sud-Est IV and by the Biomedical French Agency (ANSM). The experiment and all of the involved procedures were performed in accordance with the relevant guidelines and regulations applied in Europe.

#### Pathologies

Nine patients suffered brain damage because of a tumour (4 cavernomas: 1 temporal lobe, 1 ventromedial orbito-frontal cortex, 2 left frontal cortex; 1 ependymoma in the occipital region; 1 glioma in the parieto-temporal cortex; 1 neuroma in the cerebellopontine angle; 2 colloid cysts in the third ventricle). Of the remaining 16 patients, 9 suffered from hydrocephalus, 6 from CSF hydrodynamic abnormalities and 1 from skull base defect. CSF extraction (location was contingent on surgery type) were collected in the subarachnoid space (N = 9), within the ventricle (N = 7) or at the end of a ventriculoperitoneal shunt tap (N = 9).

#### Sampling procedures

Blood and CSF were simultaneously collected during surgery at the Neurological Hospital in Lyon. Patients fasted overnight. Seven millilitres of blood were collected in EDTA tube and up to five millilitres of CSF were collected in a polypropylene tube, immediately after CSF aspiration by the neurosurgeon. Tubes were immediately placed on ice and transported to the nearby Neurobiotec Center (Neurological Hospital, Lyon), where samples centrifugation and storage occurred. CSF and blood were centrifuged during 10 minutes at 2000 × g at 4 °C. Note that there is no need for CSF centrifugation, but this was done so all samples undergo the same treatment. Plasma and CSF were then stored in a freezer at −80 °C until assay occurred within an hour after sampling.

### Non-human primate experiment

#### Sampling procedure

Three male rhesus macaques (mean age 4.1 years), implanted for another experiment^29^ with a head post and a chamber located above the right lateral ventricle were involved in the present experiment. We precisely localized the right lateral ventricle, guided by structural MR images, and sampled 250 µL of CSF with a 23 Gauge needle and a 1 mL syringe. This procedure was done in both awake animals under head restraint conditions (7 samples) and on anesthetized animals (5 samples). For procedure details, see Lefevre and colleagues^29^. Simultaneously, 1 mL of blood was withdrawn from the saphenous vein. Immediately after collection, samples were centrifuged during 10 minutes at 2000 × g at 4 °C, and stored at −80 °C until assay. The experiment and all of the involved procedures were performed in accordance with the relevant guidelines and regulations (European Community standards for the care and use of laboratory animals) and approved by the animal ethical committee CELYNE n°42 (ref 02075-01) and the French Ministry of agriculture and environment.

### Sample and data processing

#### Oxytocin assay

Three different methods were used to measure OT in human samples, and one for macaques’ samples. They will be referred as, respectively, OT ELS (Enzo Life Science), OT Lg (the method from Geenen’s lab in Liège^28^), OT RIA (the method from Landgraf’s lab^18^), OT M (macaques).

First, we performed in our laboratory an analysis without extraction nor filtration using an Enzyme Linked-Immunosorbent Assay (EIA) kits (Enzo Life Science® Farmingdale, NY; ref ADI-900–153). It should be noted that extraction is “strongly recommended” by the manufacturer and that we deliberately chose to skip this step-in order to demonstrate the importance of the procedure on OT values. This EIA is a competitive assay (as opposed to the “sandwich” technique), and is therefore sensitive to the various interfering products in samples. Each sample was tested in duplicate (intra plates cv <10%, inter plates cv <20%). Prior to assay, plasma samples were diluted to 1:4 (concentrations values reported hereafter are the true concentrations, not the diluted ones). Cross-reactivity with related peptides is according to manufacturer <0.001% and assay sensitivities 11.6 pg/ml. We will refer to this technique as OTc (CSF) ELS and OTp (plasma) ELS.

Second, we sent our samples to an independent lab (Centre of Immunology and Neuroendocrinology of Liège University, Belgium) blind to the goal of the study, where they assayed OT using a method previously described by Péqueux *et al*.^28^ and already reported in several studies^30–32^. Briefly, this method is a laboratory developed EIA involving a filtration protocol before the assay in order to remove all molecules (I. e., plasma proteins) that weight more than 3 kDa and with a sensitivity of 1 pg/ml. Thus, samples bellow 1 pg/ml were truncated at 1 pg/ml. We will refer to this technique as OTc Lg and OTp Lg (“Lg” stands for “Liège”).

Third, we sent our samples to another independent lab headed by Pr Rainer Landgraf, who has developed the Radioimmunoassay (RIA) procedure for OT. This method has often been used to assay OT in various species and details can be found in^18^. Prior to the assay, samples were extracted as previously described in the literature^18^. The analysis was performed on codified samples, without knowing which CSF samples matched to plasma samples. We will refer to this technique as OTc RIA and OTp RIA.

Finally, monkey samples were assayed using the same EIA kit (Enzo Life Science) that was used for human samples, but with the addition of a solid phase extraction (SPE). 250 µL of 0.1% trifluoroacetic acid (TFA-H_2_O) and 250 µL of plasma were centrifuged at 16000 g for 15 minutes at 4 °C. The supernatant was applied to a 200 mg Sep Pak column (previously equilibrated with 1 mL of acetonitrile and 15 mL of 0.1% TFA- H_2_O) and washed with another 15 mL of 0.1% TFA- H_2_O. The sample was then eluted with 3 mL of 95% acetonitrile/5% of 0.1% TFA- H_2_O, and the eluate (kept cold in an ice bath) was evaporated with compressed nitrogen gas and stored at −20 °C until assay. Prior to assay, samples were reconstituted with 250 µL of assay buffer. One sample was spiked with 500 pg of OT and found to have a recovery rate of 102.5%. We will refer to this technique as OTc M and OTp M.

#### Statistical Analysis

All methods generated data having non-Gaussian data distribution (biased towards the low values). We applied log transform to all variables in order to restore normal distribution and to perform parametrical statistical tests. Pearson’s correlation tests were used to evaluate correlation between log transformed OT concentration of simultaneous CSF and blood samples. Moreover, we also tested correlations between methods for both CSF and plasma OT values.

To complement this analysis, we also used Spearman rank correlation tests on raw values, as non-parametric statistics are not sensitive to biased distribution and outliers.

Our sample size of 22–25 patients (depending on the assay) has 80% power (20% chance to fail rejecting the null hypothesis) to detect r = 0.57–0.54. We consider however that correlation coefficient lower than 0.5 would not be strong enough to use plasma OT as a reliable indicator of central OT.

All statistical tests were performed with STATISTICA 8 or MATLAB 2013a.

For cases of non-significant correlations between the methods, we used equivalence tests based on the two-one sided test procedure^33^ to determine if we could conclude statistical equivalence for the correlation between methods. We set the equivalence bounds to r = −0.5 and 0.5 as we estimate that lower coefficients of correlation are not indicating equivalent measures of the same samples by the different methods.

#### Data availability

The datasets generated and analysed during the current study are available from the corresponding authors on reasonable request.

## Results

### Raw results

Out of the 25 patients involved, we could not obtain enough CSF for 3 of them to perform every measurement method. Thus only 22 simultaneous CSF and plasma samples were measured with the OT Lg and OT RIA methods. In addition, one CSF sample could not be assessed with the OT ELS methods due to technical issue. Table 1 describes the raw concentrations obtained and the number of samples assayed with each method (for a graphical representation of all values, see Supplementary Figure 1).

**Table 1:** Raw values of OT concentrations obtained with each method.

	N	Mean	SD	Min	Max
OTc RIA	22	5.7	3.3	2.3	15.9
OTp RIA	22	4.9	2.5	2.0	10.1
OTc Lg	22	2.1	0.6	1.0	3.3
OTp Lg	22	1.3	0.3	1.0	2.0
OTc ELS	24	12.4	8.2	3.1	32.2
OTp ELS	25	1487.2	1389.0	75.3	5568.6
OTc M	12	16.2	7.0	6.4	27.2
OTp M	12	18.8	7.4	10.8	34.1

All values are in pg/ml (except N). N = number of samples, SD = Standard Deviation, Min = minimum and Max = maximum, RIA = RadioImmunoassay with extraction^18^, Lg = laboratory Enzyme Immunoassay with filtration^28^, ELS = commercial EIA (Enzo Life Science) without extraction, M (on monkey samples) = commercial EIA (Enzo Life Science) with extraction, c = CSF and p = plasma.

### Central and peripheral oxytocin (OT) concentration

**Figure 1:**
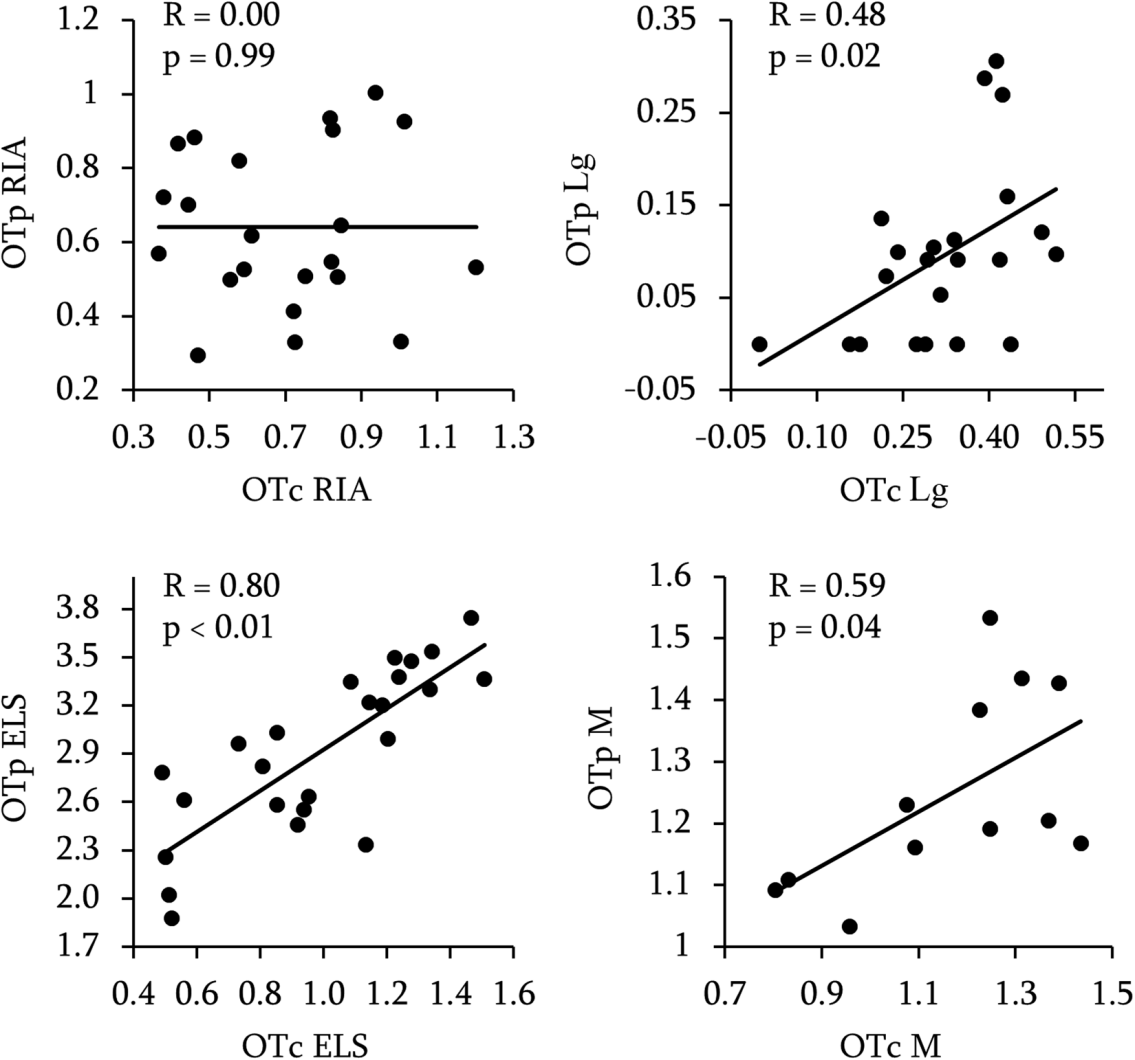
Correlation between OT concentration in the CSF (OTc, horizontal axis) and in the plasma (OTp, vertical axis). Data are log-transformed. R = Pearson’s correlation coefficient and p = p-values.

Our analysis on log transformed concentrations revealed significant positive correlations between OT concentration in the plasma and OT concentration in the CSF for three out of the four methods used, namely, OT ELS (r = 0.80, p < 0.01, 95% CI [0.59, 0.91]), OT Lg (r = 0.48, p = 0.02, 95% CI [0.06, 0.75]), OT M (r = 0.59, p = 0.04, 95% CI [0.03, 0.87]) (Fig. 1). Thus, only OT RIA did not lead to a significant correlation (r = 0.00, p = 0.99, 95% CI [−0.42, 0.42]). Correlation coefficients and p-values of Pearson’s tests are displayed on Fig. 1.

Importantly, the non-parametric analysis performed on raw values gave identical results (see Table 2 for exact values).

**Table 2:** Correlation between OT concentration in the CSF (OTc) and in the plasma using non-parametric test (Spearman) on raw values.

	RIA	Lg	ELS	M
Spearman’s Rho	0.03	0.47	0.80	0.61
P-value	>0.05	<0.05	<0.05	<0.05

### Between methods correlation

**Figure 2:**
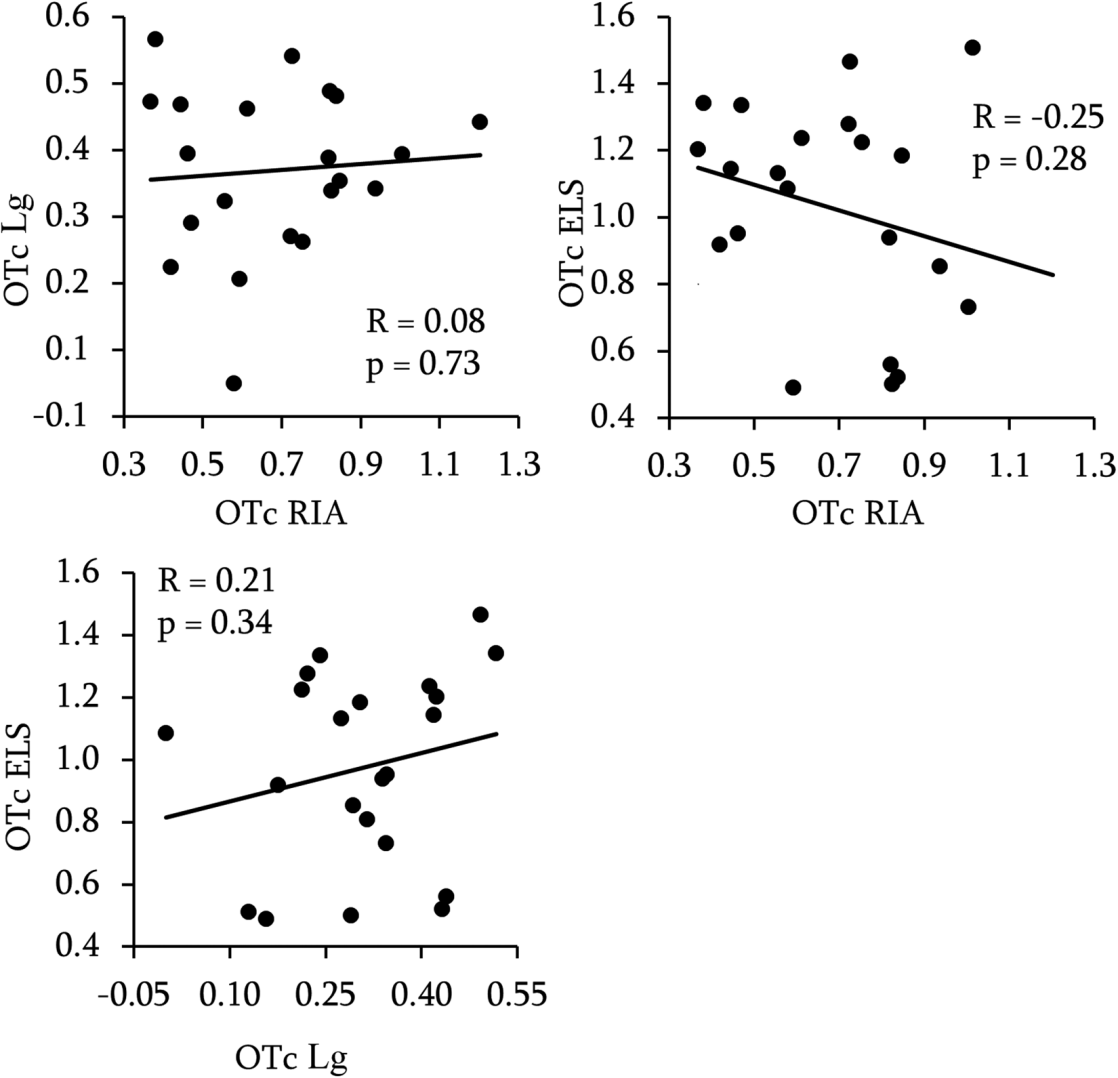
Between methods correlation of OT concentrations in the CSF. Data are log-transformed. R = Pearson’s correlation coefficient and p = p-values.

**Figure 3:**
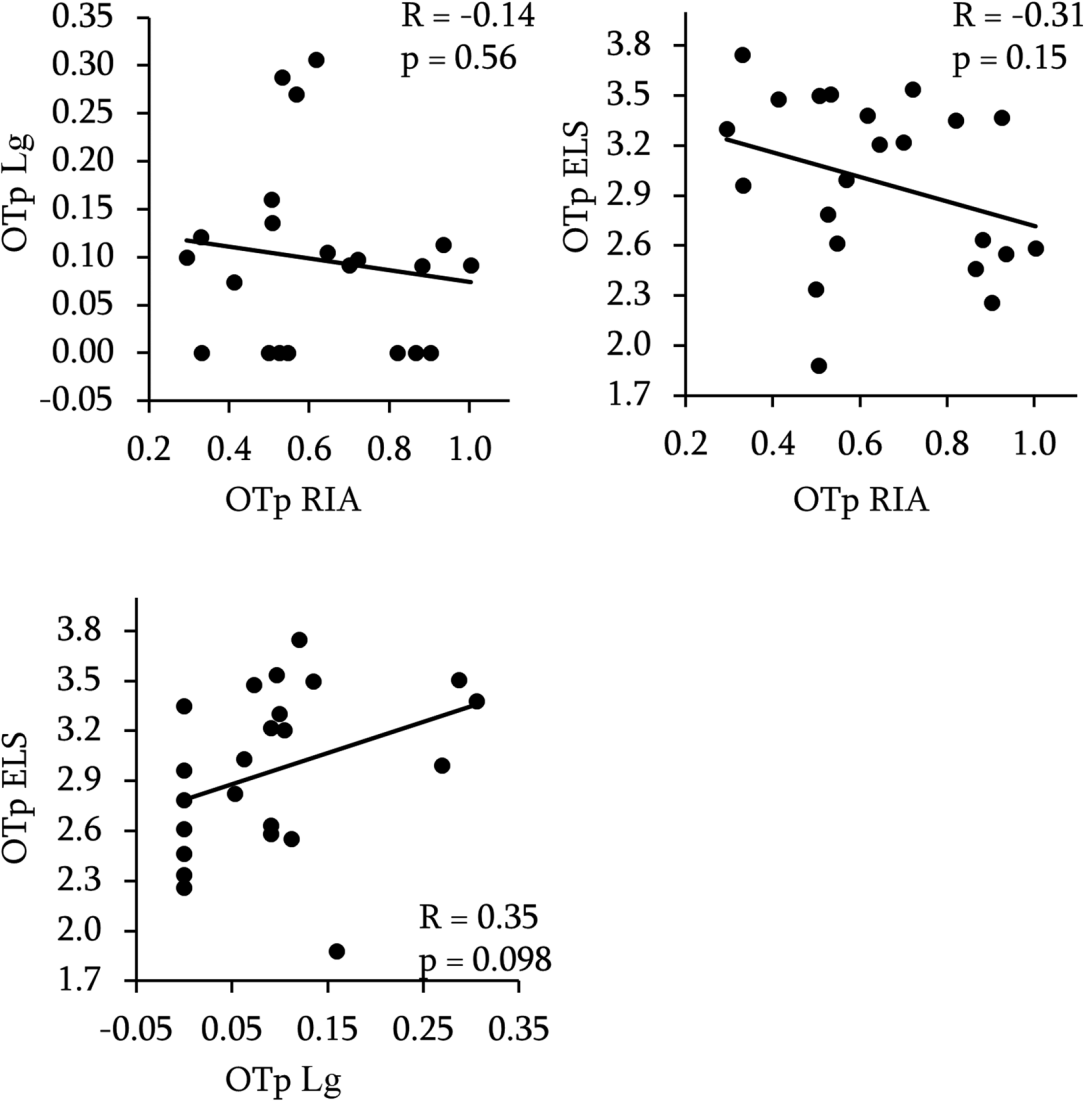
Between methods correlations of OT concentrations in the plasma. Data are log-transformed. R = Pearson’s correlation coefficient and p = p-values.

For both CSF (OTc) and plasma (OTp) OT concentrations, none of the correlations we performed revealed an effect, whether we used Pearson (CSF: RIA/Lg: r = 0.08, p = 0.73, 95% CI [−0.37, 0.49]; RIA/ELS: r = −0.25, p = 0.28, 95% CI [−0.61, 0.21]; Lg/ELS: r = 0.21, p = 0.34, 95% CI [−0.24, 0.58]; Plasma: (RIA/Lg: r = −0.14, p = 0.56, 95% CI [−0.54, 0.31]; RIA/ELS: r = −0.31, p = 0.15, 95% CI [−0.65, 0.12]; Lg/ELS: r = 0.35, p = 0.098, 95% CI [−0.07, 0.67])) or Spearman tests on log transformed (CSF, Fig. 2 and plasma, Fig. 3) or raw data (Table 3), respectively.

**Table 3:** Between methods correlation of OT concentrations in the CSF and in the plasma using non parametric test (Spearman) on raw values. All p-values > 0.05.

CSF	RIA	Lg
Lg	0.00	
ELS	−0.42	0.20
Plasma		
Lg	−0.11	
ELS	−0.35	0.35

Equivalence tests with equivalence bounds set to r = 0.5 revealed that CSF, RIA and Lg led to independent measures (p = 0.02), ELS and Lg tended to be independent (p = 0.07), and RIA and ELS were not independent (0.1) but the negative correlation of r = −0.25 is highly unexpected and does not suggest in our view an agreement between the two methods.

Equivalence tests for measurements in plasma revealed that RIA and Lg led to independent measures (p = 0.04), ELS and RIA were not independent (p = 0.16) but once again negatively correlated (r = −0.31) and ELS and Lg measures were not independent (p = 0.21).

To sum up, it can be concluded that we did not found any concordance among the methods tested, excepted for OTp ELS and RIA, for which our data are not sensitive enough to reject the null hypothesis or to support statistical equivalence.

### Effect of other variables on plasma and CSF oxytocin (OT) in humans

For each method, we did not find an effect of sex on OT levels in both CSF and plasma (all p > 0.1, see Supplementary Table S1).

Furthermore, OT levels did not correlate with any of the following variables: age, height and weight, samples freezing duration and freezing delay (time between sampling and freezing after centrifugation and aliquot) (all p > 0.1, see Supplementary Table S2).

Because OT varies throughout the day^34^ we used Kruskal-Wallis ANOVA to examine whether sampling time had an effect on OT concentrations (values were split in 3 groups according to time of sampling: between 9 am and 12 pm: n = 10, between 12 pm and 2 pm: n = 5 and between 2 and 7 pm: n = 10). No significant effect was found (p > 0.1, see Supplementary Table S3).

We also investigated whether the site of CSF extraction (constrained by patients’ pathology: ventricles (n = 9) for hydrocephaly, subarachnoid cavity (n = 7) for tumours, ventriculoperitoneal shunt tap (n = 9) for CSF hydrodynamic disorders), had an impact on OT concentration^21^. Kruskal-Wallis ANOVA analysis did not reveal any significant differences (all p > 0.1, see Supplementary Table S3).

We also tested the influence of different analgesic drugs (Remifentanil = 7, Sufentanil = 18). We found no effect (p > 0.1, see Supplementary Table S3).

Finally, in macaque monkeys, for both OTc and OTp we did not find differences between samples taken with anaesthesia compared to those taken without (Mann-Whitney U tests: Z = 0.57, p > 0.1 for CSF and Z = −0.41, p > 0.1 for plasma).

## Discussion

Our study provides two main results. First, we found a significant relationship between OTp and OTc with three methods (OT ELS, OT Lg and OT M) but the one measure considered to be the gold standard for OT measurement (OT RIA) did not led to similar results. These findings therefore call for more caution when considering what information is inferred from peripheral OT levels. Second, we found no correlations between any of the measures. The lack of agreement between OT ELS without extraction, OT Lg and OT RIA raises doubts on exactly what is measured by these methods, at least in human subjects under stressful conditions.

Given these divergent results on the relation between OTp and OTc, it appears clear that OTp cannot be used as a biomarker of central OT as its biological significance remains unclear. Aside from its well-known action in giving birth and lactation^1^, OT has also many other physiological roles, such as in pain regulation^35^, skeletal homeostasis^36,37^, muscles maintenance and regeneration^38^, energy metabolism^39^, body temperature^40,41^, cardiovascular monitoring^42–44^. It must be noted that while pain seems to be jointly regulated by OTc and OTp, it is less clear if other peripheral effects of OT are linked to central OT, even though this could still occur through peripheral feedback to the brain^45,46^. Thus, even if OTp and OTc are correlated under specific circumstances, the information given by these two measures is unlikely the same. Hence, correlating OTp with behavioural scales seems to be a suboptimal method to investigate links between the OT system and behaviour, as OTp might be a noisy proxy of OTc. Of course, it can be hypothesized that physiological actions of OTp in the body are synchronized to OTc effects on behaviour, for instance to adapt cardiac rhythm to the emotional state^44,47^. But this assumption still need to be demonstrated.

Adding to this problem is the absence of agreement between methods tested here. Importantly, while this issue was expected for OT plasma concentration given the influence of pre-processing procedure (none, filtration, extraction), the fact that CSF OT levels assayed with three different specific antibodies were completely unrelated (as shown by the equivalence analysis) is surprising, as there are supposedly no interfering proteins in this matrix. One can expect different concentrations values due to dissimilar assay sensitivity, but the order between values should remain identical. This was not the case here, as even non-parametric tests did not lead to significant results.

Thus, more work is necessary to compare OT methods and one important factor is sample pre-processing^25,27^. A recent finding showing that in the blood OT can bind to a larger molecule^48^ may explain the differences we observed here, suggesting that after extraction or filtration we might only assay “free OT” but ignore the OT that is bound. This hypothesis leads to new questions regarding the biological significance of “free OT” and “bound OT”, the rate of exchange between the two compartments and if the OT we measure without extraction or filtration corresponds to the combined free and bound OT. One critical step will be the identification of the molecule(s) to which OT binds to, and whether several potential candidates such as albumin or Neurophysin 1 can be considered^49,50^. Unfortunately, this idea is still very speculative and deserves confirmation and further research.

We have no recommendations here regarding the optimal way to assess OT levels, as these issues should be clarified by scientists and companies providing these methods. We believe that in depth chemical analysis is required to check and compare the influence of pre-processing methods on endogenous OT and if differences in binding site recognition by the various antibodies could be responsible for such discrepancies. Recently, several teams have used liquid chromatography coupled to tandem mass spectrometry (LCMSMS) to assess OT levels^35,51,52^. They all reported plasma OT levels, following extraction, within the classical range of 1–10 pg/ml, (but see^48,53^). We suggest that it would be interesting to look at the effect of pre-processing on these values, and whether OT levels measured in the CSF with LCMSMS are coherent with those measured by EIA and RIA.

Our experiment has some limitations. We extracted CSF and blood at least 30 minutes after general anaesthesia and skin incision. We assumed this was an optimal interval to observe a similar modulation in plasma OT and CSF OT concentrations contrary to lumbar puncture procedure, which causes acute stress, and therefore OT release may not have enough time to act on CSF OT concentration. In addition, lumbar puncture generates pain which probably prompts a fast release of oxytocin in some segments of the spinal cord^23^. Lastly, CSF flow is much slower in spinal cord than in central system^7^, so CSF OT at lumbar level can be reduced. Altogether, we believe that CSF OT levels obtained after lumbar puncture might be too variable.

It should be noted that due to limited volume of samples in humans, we were not able to perform extraction prior to assay with the commercial EIA (ELS). Notably, the manufacturer strongly recommends this step and therefore we must acknowledge that this might have increased the disagreement between the methods tested here. Thus, from the present results we cannot conclude whether ELS with extraction correlates with the other methods.

Our patients were suffering from different types of pathologies (tumour, hydrocephalus or CSF hydrodynamic disorder) but no differences in the hormones’ levels were observed between the three groups. This can be explained by the absence of damage in the hypothalamus, the core area of oxytocin synthesis. Moreover, the wide variety of patients’ aetiology indicates that our results are unlikely due to a specific pathological effect.

The various anaesthetics and analgesic drugs used during surgery did not produce any effect on the neuropeptide concentrations as well, and this result is in accordance with a previous study showing that, during surgery, plasma levels of OT raised after skin incision but not after anaesthesia induction^54^. Furthermore, our results in non-human primates also show that anaesthesia does not alter OT levels.

In our equivalence analysis, because of our sample size, we had to set equivalence bounds to r = −0.5 and 0.5. Thus, we could not reject smaller correlations. However, correlations lower than 0.5 would still, in our opinion, be insufficient to use plasma OT as a reliable marker of its action in the brain. Future studies are needed to clarify this point.

Following assay of plasma with the OT Lg technique, several values (n = 7) had to be truncated to 1 pg/ml. It should be noted that higher sensitivity could have impacted the correlation between plasma OT and CSF OT, and therefore this specific result should be interpreted with additional caution.

Finally, although we found a correlation between OTc and OTp with the commercial EIA after extraction, this result was obtained in a limited number of samples (n = 12) from rhesus macaques, and we cannot rule out potential species differences with humans.

Altogether, we believe that our protocol produced a release of OT at both peripheral and central level, given the intensity and the length of stress induced by a surgical intervention. Thus, it should be noted that, given the specific context in which we collected our data, we cannot firmly conclude on the link between OTc and OTp at basal state. Our study is in agreement with a recent review^24^ suggesting that OTp and OTc might be correlated under specific stress conditions although this seems to vary with the method employed. Therefore, we believe that plasma OT should not be used to infer central OT levels, unless one can prove that in the sampling conditions, and with the assay method used, plasma OT is correlated to central OT. Last but not least, we call for more inter methods comparisons in both plasma and CSF (as well as saliva and urine) samples, as it seems that each method is producing different values of OT concentrations.

## Electronic supplementary material

supplementary results
