# Supplementary material for: A comparison of methods to measure central and peripheral oxytocin concentrations in human and non-human primates
Source: Sci Rep. 2017 Dec 8;7:17222. doi: 10.1038/s41598-017-17674-7 (PMC5722864; doi:10.1038/s41598-017-17674-7)
Supplement: Supplementary file 1 — supplementary results [file 41598_2017_17674_MOESM1_ESM.pdf]

Supplementary results

A comparison of methods to measure central and peripheral oxytocin concentrations in human and non-human primates

Arthur Lefevre[1,2,*], Raphaëlle Mottolese[1,2], Manon Dirheimer[1], Carmine Mottolese[1,3], Angela Sirigu[1,2]

[1] Institut des Sciences Cognitives Marc Jeannerod, UMR 5229, Centre National de la Recherche Scientifique, Bron, France.

[2] Université Claude Bernard Lyon 1, Lyon, France.

[3] Neurosurgery Unit 500, Hôpital Pierre Wertheimer, Hospices Civils de Lyon, Lyon, France.

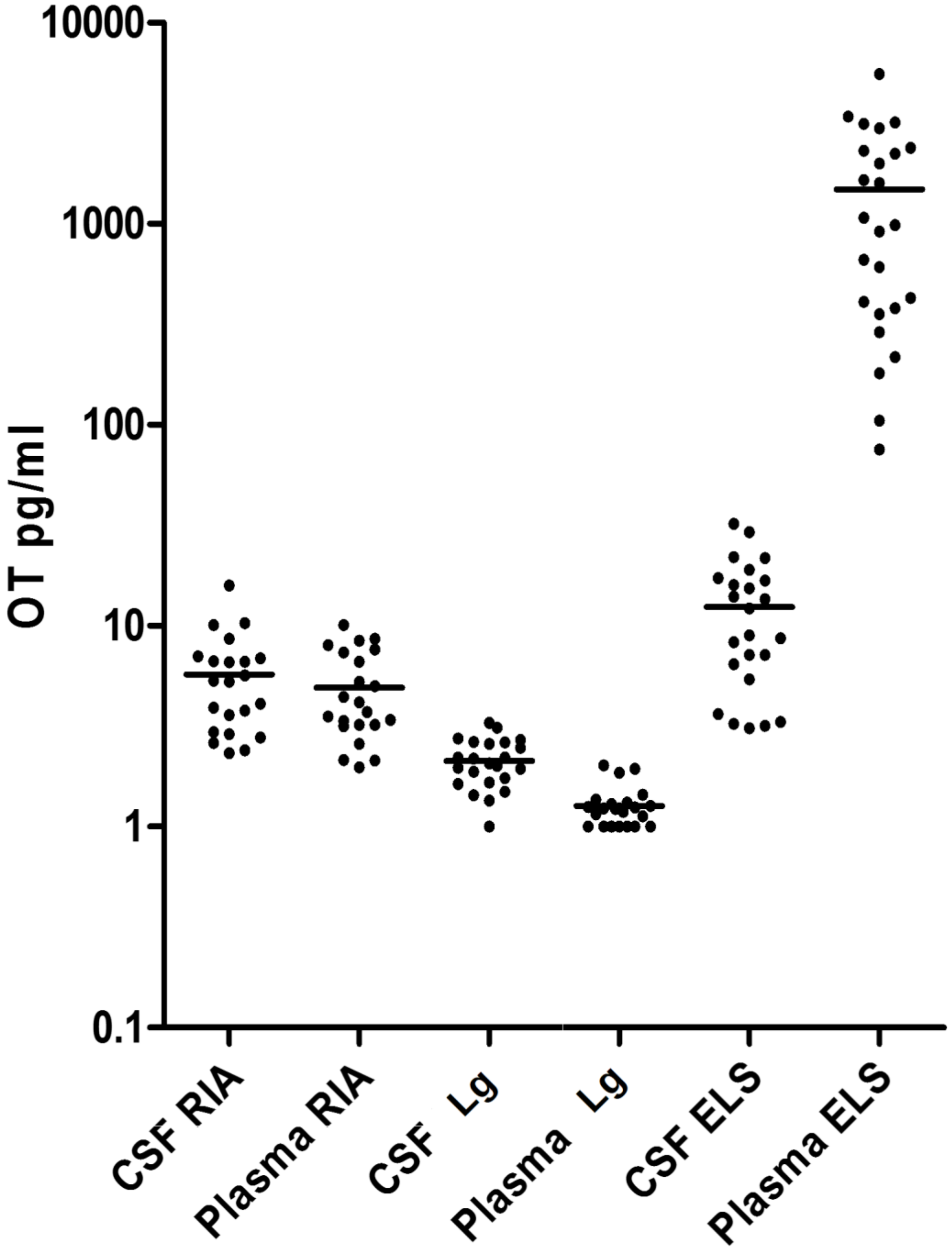

Figure S1. Individual values of OT concentration on a logarithmic scale for each CSF and plasma samples assayed with the 3 methods. Black bars represent the mean.

Table S1. Mean and standard deviations of plasma and CSF OT concentrations obtained with the three different methods for females and males. Mann Whitney U tests Z statistic and p values (Bonferroni corrected for multiple comparisons, p*6).

		CSF RIA	Plasma RIA	CSF Lg	Plasma Lg	CSF ELS	Plasma ELS
Females	Mean	6.48	5.41	2.10	1.40	15.10	1719.06
	SD	3.83	2.89	0.52	0.37	9.48	1599.94
Males	Mean	4.86	4.32	2.16	1.15	9.71	1235.93
	SD	2.43	1.76	0.66	0.16	6.02	1133.38
Mann Whitney	Z	0.99	0.86	-0.49	1.88	1.41	0.38
	P corrected	> 0.1	> 0.1	> 0.1	> 0.1	> 0.1	> 0.1

Table S2. Pearson coefficients of correlation between OT concentrations and confounding variables. Delay refers to the time between sampling and storage to -80°. Storage refers to the duration of storage. None of them reached statistical significance (all p > 0.1, corrected p = p*6).

	CSF RIA	Plasma RIA	CSF Lg	Plasma Lg	CSF ELS	Plasma ELS
Age	0.34	-0.12	-0.09	0.39	0.34	0.50
Height	-0.27	-0.32	0.32	-0.39	-0.33	-0.18
Weight	-0.08	0.25	0.16	-0.37	-0.14	-0.29
Delay	0.03	0.01	0.40	0.00	-0.28	-0.31
Storage	-0.03	-0.31	0.15	0.23	0.53	0.55

Table S3. Effects of confounding factors anaesthetics, time of sampling and location of sampling on OT concentrations, analysed with Mann Whitney U test (Z) or Kruskall Wallis ANOVA (H). All p > 0.1.

		CSF RIA	Plasma RIA	CSF Lg	Plasma Lg	CSF ELS	Plasma ELS
Anaesthetics	Z	-0.83	-0.04	1.07	-0.50	-0.43	-0.82
Time	H	2.17	1.54	6.31	6.18	2.66	3.02
Location	H	3.01	4.80	1.02	1.40	1.50	0.78
